# Plant Regeneration *via* Somatic Embryogenesis in Mature Wild Olive Genotypes Resistant to the Defoliating Pathotype of *Verticillium dahliae*

**DOI:** 10.3389/fpls.2019.01471

**Published:** 2019-11-14

**Authors:** Isabel Narváez, Carmen Martín, Rafael M. Jiménez-Díaz, Jose A. Mercado, Fernando Pliego-Alfaro

**Affiliations:** ^1^Instituto de Hortofruticultura Subtropical y Mediterránea “La Mayora” (IHSM-UMA-CSIC), Departamento de Botánica y Fisiología Vegetal, Universidad de Málaga, Málaga, Spain; ^2^Departamento de Biotecnología-Biología Vegetal, ETS Ingeniería Agronómica, Alimentaria y de Biosistemas, Universidad Politécnica de Madrid, Madrid, Spain; ^3^Departamento de Agronomía, College of Agriculture and Forestry (ETSIAM), Universidad de Córdoba, Campus de Excelencia Internacional Agroalimentario ceiA3, Edificio C-4 Celestino Mutis, Campus Rabanales, Ctra. de Madrid, Córdoba, Spain

**Keywords:** somatic embryo, oleaster, adult explants, embryo conversion, Verticillium wilt

## Abstract

Regeneration capacity, *via* somatic embryogenesis, of four wild olive genotypes differing in their response to defoliating *Verticillium dahliae* (resistant genotypes StopVert, OutVert, Ac-18 and the susceptible one, Ac-15) has been evaluated. To induce somatic embryogenesis, methodologies previously used in wild or cultivated olive were used. Results revealed the importance of genotype, explant type, and hormonal balance in the induction process. Use of apical buds obtained from micropropagated shoots following a methodology used in cultivated olive (4 days induction in liquid 1/2 MS medium supplemented with 30 µM TDZ–0.54 µM NAA, followed by 8 weeks in basal 1/2 MS medium) was adequate to obtain somatic embryos in two genotypes, StopVert and Ac-18, with a 5.0 and 2.5% induction rates, respectively; however, no embryogenic response was observed in the other two genotypes. Embryogenic cultures were transferred to basal ECO medium supplemented with 0.5 µM 2iP, 0.44 µM BA, and 0.25 µM indole-3-butyric acid (IBA) for further proliferation. Somatic embryos from StopVert were maturated and germinated achieving a 35.4% conversion rate. An analysis of genetic stability on StopVert, using Simple Sequence Repeats (SSRs) and Random Amplified Polymorphic DNA (RAPDs) markers, was carried out in embryogenic callus, plants regenerated from this callus and two controls, micropropagated shoots used as explant source, and the original mother plant. Polymorphism was only observed in the banding pattern generated by RAPDs in 1 of the 10 callus samples evaluated, resulting in a variation rate of 0.07%. This is the first time in which plants have been regenerated *via* somatic embryogenesis in wild olive.

## Introduction

The cultivated olive *Olea europaea* L. subsp. *europaea* var. *sativa*, Oleaceae family, is a long-lived, evergreen medium-size tree, adapted to dry and poor soils. Fruits have high nutritional value due to their high lipid content. Olive is one of the most important oil crops within the Mediterranean basin (Rugini, 1995). This region accounts for over 96% of the 11.4 million ha of olive trees cultivated worldwide (Rallo et al., 2018). In 2017, total olive oil production was ca. 2,881 thousand tons, with Spain, Italy, Greece, and Portugal as more relevant producing countries (IOC, International Olive Council, 2018).

Wild olive (*O. europaea* L. subsp. *europaea* var. *sylvestris*) is considered as the main progenitor of cultivated olive since both have the same ploidy level (2n = 2x = 46) and similar morphological traits and environmental requirements (Besnard and Rubio de Casas, 2016). Wild genotypes could be useful germplasm sources in olive breeding for introducing resistance to biotic (Colella et al., 2008) or abiotic stress (Murillo et al., 2005). Additionally, in recent years, there has been an increasing interest in the use of pathogen-resistant selections of wild olive as rootstocks to reduce the negative impact of some diseases, especially Verticillium wilt (Jiménez-Fernández et al., 2016). This disease, caused by the soil-borne pathogen *Verticillium dahliae* Kleb., is considered as the main threat to olive production worldwide (Jiménez-Díaz et al., 2012). Most economically important cultivated genotypes are susceptible or extremely susceptible to this disease (López-Escudero et al., 2004). Some wild olive selections highly resistant to defoliating *V. dahliae* pathotype have been identified and used to develop a grafted commercial product, Vertirés, currently available to growers (Jiménez-Díaz, 2018; Jiménez-Díaz and Requena, 2018).

Although olive is generally difficult to manipulate *in vitro*, it has been possible to micropropagate selected olive cultivars through nodal segmentation of elongated shoots (Rugini, 1984; Roussos and Pontikis, 2002; Lambardi et al., 2013). In few cases, buds (Bahrami et al., 2010) or plants (Mencuccini and Rugini, 1993) have been obtained through adventitious organogenesis from petiole and leaf sections derived from *in vitro* grown shoots of adult origin. However, the most widely used method for adventitious regeneration in both cultivated and wild olive is somatic embryogenesis, although, in this case, most investigations have been carried out with juvenile material, i.e., either immature zygotic embryos (Rugini, 1988) or radicle and cotyledon segments from mature embryos (Orinos and Mitrakos, 1991; Mitrakos et al., 1992; Cerezo et al., 2011). Regarding adult material, Rugini and Caricato (1995) developed a double regeneration system, using petioles derived from shoots of adventitious origin as explants, to obtain somatic embryos and plants from the Italian cvs. Canino and Moraiolo. Other authors used leaf and petioles isolated from *in vitro* shoots of cultivars Dahbia (Mazri et al., 2013) and Picual (Toufik et al., 2014). In wild olive, self-rooted plants in greenhouse were an adequate source of explants; however, plant regeneration was not reported (Capelo et al., 2010).

Alterations originating during *in vitro* culture, referred as somaclonal variation (SV) (Larkin and Scowcroft, 1981), are one of the main drawbacks of *in vitro* techniques for clonal propagation or plant regeneration of elite germplasms. These variations can occur due to several factors, i.e., *in vitro* propagation method, genotype, type of explant, growth regulator type and concentration, time in culture, as well as stress generated during the *in vitro* phase (Bairu et al., 2011). In general, micropropagation through axillary branching seems to be quite reliable in terms of genetic stability (George and Debergh, 2008), while indirect somatic embryogenesis is considered to be a genetically unstable process, especially when material has been kept for prolonged time in culture (Vázquez, 2001; Bradaï et al., 2016). The balance and type of growth regulators affect the frequency of occurrence of SV; in fact, high levels of auxins and cytokinins induce changes in DNA methylation patterns (LoSchiavo et al., 1989) or ploidy levels (Bouman and De Klerk, 2001). Hence, somatic embryogenesis protocols should be assessed for their effects in the obtainment of true-to-type plants. To examine genetic stability several molecular markers have been recommended, being microsatellites or Simple Sequence Repeats (SSRs) and Random Amplified Polymorphic DNA (RAPDs) the most commonly used (Bairu et al., 2011). RAPD markers are considered less reproducible than SSRs; however, both methods have been employed in olive to ascertain genetic stability of *in vitro* material (García-Férriz et al., 2002; Doveri et al., 2008; Leva and Petruccelli, 2012).

The main objective of this study was to evaluate the embryogenic capacity of different explants of adult origin, from *V. dahliae*-resistant and susceptible genotypes, of wild olive. Afterwards, genetic stability of embryogenic callus as well as plants regenerated from somatic embryos was evaluated by using SSRs and RAPD markers.

## Materials and Methods

### Plant Material and Culture Establishment

*In vitro* shoots from adult plants of wild olive genotypes highly resistant (namely Ac-18, OutVert and StopVert) or susceptible (namely Ac-15) to D *V. dahliae* (Colella et al., 2008; Jiménez-Fernández et al., 2016) were used as explants source. Shoot cultures had been established from lateral buds of self-rooted mother plants, grown in the greenhouse (**Supplementary Figure 1**), and were maintained on RP proliferation medium [DKW macro and micronutrients as modified by Roussos and Pontikis (2002), vitamins of Roussos and Pontikis (2002)] with a 2 mg/L zeatin riboside supplement (Vidoy-Mercado et al., 2012). Subculturing was carried out at 6–8 week intervals.

### Somatic Embryogenesis Induction

To induce somatic embryogenesis, different types of explant were used: shoot apex with emerging leaf primordia (1.5–2 mm), the first pair of developing leaves without petiole (3–4 mm), the basal part of the following pair of developing leaves (4–5 mm), and petioles from this pair of leaves (1 mm). Leaves were cultured on the medium with adaxial side up.

Initially, and following the protocol of Capelo et al. (2010) for mature wild olive, explants from Ac-18 and Ac-15 genotypes were cultured on MS induction medium supplemented with 12.25 µM indole-3-butyric acid (IBA)–4.56 µM zeatin (Zea) for 3 months. Afterwards, calli were transferred to MS medium without growth regulators for 12 weeks. Subculturing was carried out at 4-week intervals.

In a different experiment, the protocol of Mazri et al. (2013) for cultivated olive was evaluated, i.e., explants of the four genotypes (Ac-18, OutVert, StopVert and Ac-15) were cultured on 5 ml liquid induction medium composed by 1/2 MS mineral elements, MS vitamins, 100 mg/L myo-inositol, 30 µM TDZ and 0.54 µM NAA, in septate Petri dishes for 4 days, over an orbital shaker (platform size 41 × 41 cm, New Brunswick Scientific, Edison, NJ), at 80 rpm. Afterwards, explants were cultured on solid 1/2 MS medium without growth regulators for two recultures of 4 weeks each. Callus formed after each reculture (4 and 8 weeks) was characterized according to color, texture, and proliferation rate, estimated as the percentage area of explant covered by callus using a rating scale from 0–3 (0: no callus; 1: 1–40% explant surface covered with callus; 2: 40–80%; 3: > 80%) (**Supplementary Figure 2**). Afterwards, isolated calli were transferred to olive cyclic embryogenesis medium, ECO medium (Pérez-Barranco et al., 2009), containing 1/4 OM macroelements (Rugini, 1984), 1/4 MS microelements (Murashige and Skoog, 1962), 1/2 OM vitamins, 550 mg/L glutamine, 0.25 µM IBA, 0.5 µM 2iP, 0.44 µM BA, and a 200 mg/L cefotaxime supplement, for further proliferation. Callus was recultured in this medium at 4-week intervals.

In callus induction experiments, 20–40 shoot apices, 40 petioles, and 40 leaf explants of each type were used. Cultures were incubated at 25 ± 2°C in darkness. All culture media were supplemented with 30 g/L sucrose and media solidified with 6 g/L agar. The pH of media was adjusted to 5.74.

To evaluate multiplication rate of embryogenic callus from StopVert and Ac-18 genotypes, 0.5 g of globular structures, < 3 mm diameter, were cultured on 25 ml solid ECO medium in darkness (Pérez-Barranco et al., 2009). Average weight increment from five Petri dishes was calculated at 4-week intervals during four subcultures.

### Maturation and Germination of Somatic Embryos

Globular somatic embryos, <3 mm diameter, from embryogenic callus of StopVert genotype were maturated according to Cerezo et al. (2011), i.e., embryos were cultured in Petri dishes containing basal ECO medium, supplemented with 1 g/L activated charcoal, for 2 months under dark. During the second month, somatic embryos were cultured onto a semipermeable cellulose acetate membrane (MW cut-off 12,000, Sigma D9777).

For germination, mature embryos were cultured on modified MS medium with 1/3 MS macroelements, MS microelements, and 10 g/L sucrose (Clavero-Ramírez and Pliego-Alfaro, 1990) for 2 recultures of 6 weeks each. Afterwards, isolated shoots were cultured on modified RP proliferation medium according to Vidoy-Mercado et al. (2012). Shoots derived from different germinated embryos were micropropagated separately. Embryo germination and shoot micropropagation were carried out under 40 µmol·m^-2^·s^-1^ light irradiance.

### Analysis of Genetic Stability

#### Plant Material

The genetic stability of the embryogenic callus derived from shoot apex of StopVert genotype, as well as plants regenerated from this callus, was evaluated by SSRs and RAPDs analyses. The embryogenic callus had been maintained for over 20 months on ECO medium with regular subcultures at 4–5 week intervals. For regenerated plants, leaf pieces from 10 independent shoots micropropagated in RP medium were examined. Two controls were used, a) leaves from micropropagated shoots, used as explant source, and b) leaves from the original donor plant maintained in the greenhouse. The micropropagated shoots had been initiated from lateral buds of the donor plant and maintained in RP proliferation medium for over 36 months. Different plant materials used, and the number of samples analyzed is indicated in **Table 1**.

**Table 1:** Plant material of StopVert genotype used for genetic stability analysis.

Plant material	Origin	Time in culture	N° of samples analyzed
Control donor plant	Rooted cutting	–	1
Control *in vitro* shoots used as explant source	Micropropagated shoots derived from axillary buds of donor plant	36 months	10
Embryogenic callus	Shoot apex isolated from *in vitro* shoots	20 months	10
Regenerated shoots	Plants regenerated from somatic embryos	20 months in callus phase + 3 months on RP proliferation medium after isolation from the embryo	10

#### DNA Extraction

For genomic DNA extraction, embryogenic callus or leaf material, 0.04–0.06 g, was powdered in liquid nitrogen using the TissueLyser II (Qiagen). In the case of the donor plant, young leaves were washed in 70% ethanol prior to powdering. DNA was extracted using the protocol of Gawel and Jarret (1991), resuspended in sterile milliQ water and treated with 1 µl of RNase (10 µg/ml) at 37°C for 2 h. DNA concentration and purity were visualized on a 0.8% agarose gel.

#### SSRs Analysis

Five SSR markers were selected to evaluate genetic stability: AJ279854, AJ279859 and AJ279867 (Sefc et al., 2000), and AJ416322 and AJ416323 (De la Rosa et al., 2002) (**Table 2**). The forward primer of each pair was labelled with a fluorescent dye (ABI dyes: 6-FAM™ or HEX™). All PCRs were carried out in a Mastercycler (Eppendorf) DNA thermal cycler. PCR was carried out following the protocol of MyTaq™ DNA polymerase (Bioline), being the final volume 20 µl. Samples contained approximately 10 ng of genomic DNA, 0.5 µM of each primer, and 1.25 U of MyTaq DNA polymerase in the buffer provided by the enzyme manufacturer with all needed PCR reagents (containing dNTPs and MgCl_2_). Amplification conditions were: 1 min at 95°C, 35 cycles of 15 s at 95°C, 15 s at the Tm of each specific pair of primers (**Table 2**), and 10 s at 72°C, with a final extension step of 10 min at 72°C. PCR products were visualized on a 1.5% agarose gel under non-denaturing conditions with ethidium bromide. Amplification products were analyzed in an automated ABI3730 sequencer by the company SECUGEN S.L. (Madrid, Spain). Results were analyzed with GeneMarker (v1.90 software, SoftGenetics, LLC).

**Table 2:** Microsatellites markers used in the genetic stability analysis of StopVert genotype.

Locus	Marker	Repeat motif	Sequence (5´-3´)	Allele size (bp)	T° annealing (°C)
AJ279854	ssrOeUA-DCA03	(GA)_19_	HEX™-CCCAAGCGGAGGTGTATATTGTTAC TGCTTTTGTCGTGTTTGAGATGTTG	228–250	50
AJ279859	ssrOeUA-DCA09	(GA)_23_	6-FAM™-AATCAAAGTCTTCCTTCTCATTTCG GATCCTTCCAAAAGTATAACCTCTC	161–205	55
AJ279867	ssrOeUA-DCA18	(CA)_4_CT(CA)_3_(GA)_19_	6-FAM™-AAGAAAGAAAAAGGCAGAATTAAGC GTTTTCGTCTCTCTACATAAGTGAC	168-184	50
AJ416322 AJ416323	EMO13 EMO30	(CT)_4_(CA)_8_ (AC)_8_	6-FAM™-AGGGTGGGGATAAAGAAGAAGTCAC TTTTACCCCATATACCCCGATTCATT HEX™-GTCTCTGCCCAACAATG CATACATGAGTGTGTGTG	118-139 183-196	60 50

#### RAPD Analysis

Five RAPD markers from Operon Technologies Inc. (Alameda/CA, USA) collection were used (A1: 5´- CAGGCCCTTC-3´; B7: 5´- GGTGACGCAG-3´; B15: 5´- GGAGGGTGTT-3´; E19: 5´- ACGGCGTATG-3´; F10: 5´- GGAAGCTTGG-3´). PCR was carried out modifying the protocol of MyTaq™ DNA polymerase, being the final volume of 25 µl. Samples contained approximately 10 ng of genomic DNA, 0.8 µM of a single decanucleotide, and 1.5 U of MyTaq DNA polymerase in the reaction buffer. Amplification conditions were: 2 min at 94°C, 35 cycles of 45 s at 92°C, 1 min at 37°C, and 2 min at 72°C, with a final extension step of 10 min at 72°C. PCR products were visualized by loading 12 µl on a 1.5% agarose gel under non-denaturing conditions. The size of the amplified bands was related by reference to the molecular size marker (100 Base-Pair Ladder, GE Healthcare). All PCRs were carried out at least in duplicate, in a Mastercycler (Eppendorf) DNA thermal cycler.

## Results

### Somatic Embryogenesis Induction

Use of the protocol described by Capelo et al. (2010) for wild olive did not yield embryogenic callus in any of the explants evaluated from Ac-18 or Ac-15 genotypes. After 4 weeks on induction medium (MS supplemented with 12.25 µM IBA–4.56 µM zeatin), explants of Ac-15 genotype showed higher callus proliferation rate than those from the Ac-18 genotype. The higher amount of callus was observed in shoot apex and first pair of leaves, while smaller calluses appeared in petiole explants. After two additional recultures in induction medium, calluses became compact, of white color, and showed a noticeable increase in size; however, some calluses of Ac-15 genotype were easily crumbled. Following transfer to basal MS medium, calluses started to get brown and roots appeared in some of those derived from shoot apex explants; however, no clear embryogenic structures could be identified.

**Figure 1:**
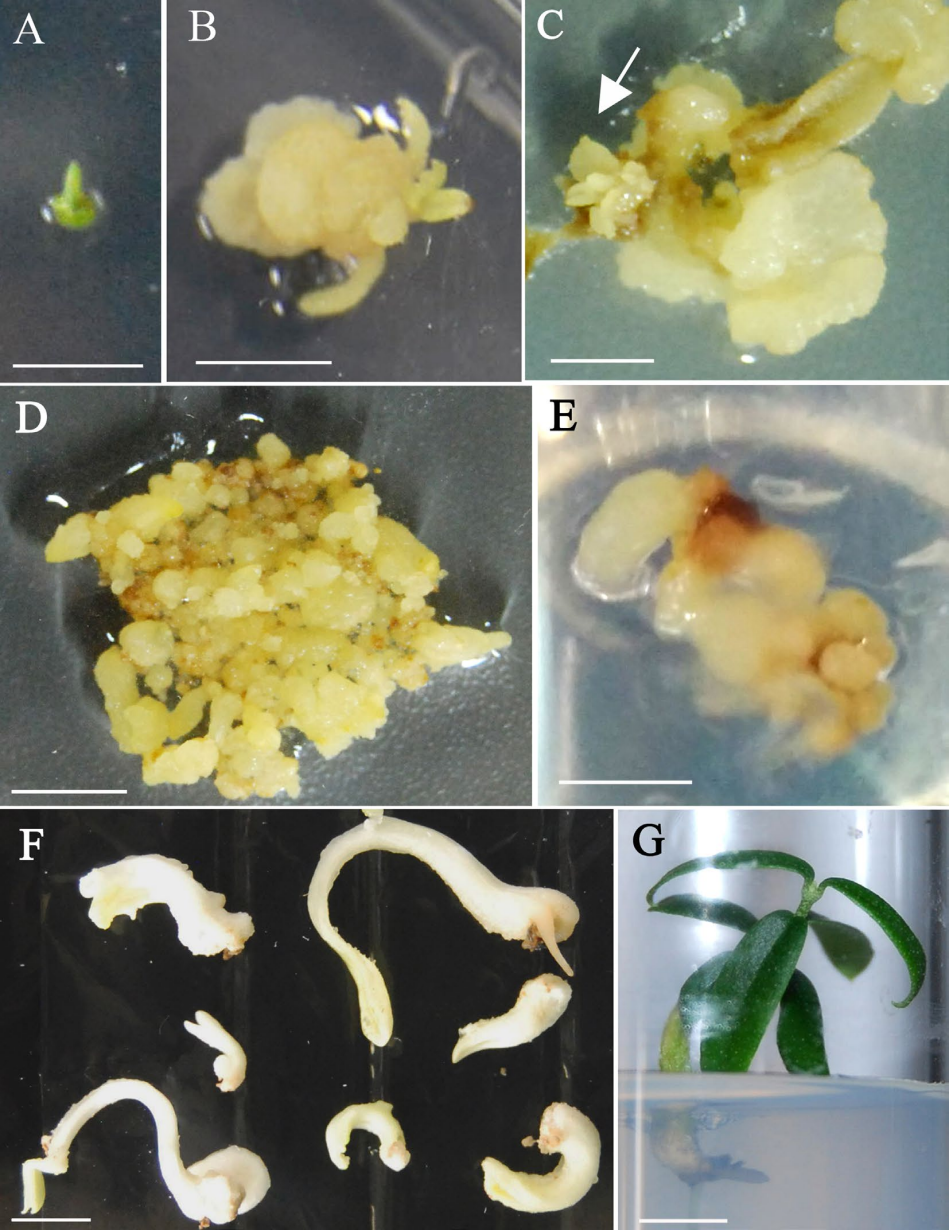
Somatic embryogenesis induction and plant recovery from adult wild olive. **(A)** Shoot apex explants after 4 days on 1/2 MS liquid medium supplemented with 30 µM TDZ–0.54 µM NAA. **(B)** Callus after 4-week culture on 1/2 MS solid basal medium. **(C)** Callus after 8-weeks culture on 1/2 MS solid basal medium with emerging embryogenic structures (arrow). Calli from StopVert **(D)** and Ac-18 **(E)** genotypes proliferating in ECO medium supplemented with 0.25 µM IBA, 0.5 µM 2iP–0.44 µM BA and 200 mg/L cefotaxime. **(F)** Somatic embryos of StopVert selection with white opaque appearance following culture on semi-permeable cellulose acetate membrane on basal ECO medium with activated charcoal. **(G)** Plant of StopVert genotype regenerated from somatic embryo after 12 weeks on germination medium. Bars correspond to 0.5 cm **(A**–**D)** and 1 cm **(E**–**G)**.

In a subsequent experiment, the protocol of Mazri et al. (2013) was assayed using the four genotypes. No noticeable changes were observed after 4 days in liquid induction medium (1/2 MS 30 µM TDZ-0.54 µM NAA) (**Figure 1A**). Calli started to form at the edges of explants after 2 weeks on solid basal medium (1/2 MS) and were clearly visible in most explants 2 weeks later (**Figure 1B**). A general increase in callus formation was observed after four additional weeks in 1/2 MS basal medium. Best response in terms of percentage of explants forming callus and amount of callus after 8 weeks of culture was observed in shoot apex explants (**Table 3**). Generally, calli were white and with a certain grade of compactness; in some cases, translucent areas were observed. Although all tested explants from the four genotypes produced callus, embryogenic structures (**Figure 1C**) were only visible in shoot apex derived callus from StopVert and Ac-18, yielding percentages of embryogenic induction of 5.0% and 2.5%, respectively (**Table 3**).

**Table 3:** *In vitro* response of different type of explants from four wild olive genotypes after 4 days induction in liquid 1/2 MS medium supplemented with 30 µM TDZ–0.54 µM NAA followed by 8 weeks culture on basal 1/2 MS medium.

Genotype	Explant	Explants with callus (%)	Amount of callus	Embryogenic callus (%)
StopVert	Shoot apex	100	2.6 ± 0.5	5.0
	Leaf (first pair)	100	1.7 ± 0.5	0
	Leaf (second pair)	64	0.7 ± 0.6	0
	Petiole	100	1.4 ± 0.8	0
OutVert	Shoot apex	100	2.0 ± 0.6	0
	Leaf (first pair)	90	1.1 ± 0.5	0
	Leaf (second pair)	87.5	1.1 ± 0.6	0
	Petiole	82	1.0 ± 0.6	0
Ac-18	Shoot apex	100	2.6 ± 0.5	2.5
	Leaf (first pair)	100	1.7 ± 0.5	0
	Leaf (second pair)	64	1.6 ± 0.5	0
	Petiole	100	2.8 ± 0.4	0
Ac-15	Shoot apex	100	3.0 ± 0	0
	Leaf (first pair)	100	1.7 ± 0.5	0
	Leaf (second pair)	100	1.5 ± 0.5	0
	Petiole	100	2.5 ± 0.7	0

The amount of callus was estimated by an arbitrary scale based on visual criteria (0: no callus; 1: < 40% explant surface covered by callus; 2: 40–80% explant surface covered by callus; 3: 80–100% explant surface covered by callus).

Embryogenic calli were isolated and cultured on ECO medium to enhance proliferation; however, while calli of StopVert performed well showing globular structures and stable growth rate throughout four subcultures (0.85 ± 0.12 g of weight increase per subculture) (**Figure 1D**), embryogenic callus from Ac-18 genotype grew poorly under these conditions (**Figure 1E**).

### Maturation and Germination of Somatic Embryos

Following the protocol of Cerezo et al. (2011), 81 globular somatic embryos from StopVert genotype were transferred to ECO maturation medium over cellulose acetate membranes and 48 of them (59.3%) changed to a white opaque appearance (**Figure 1F**). Afterwards, these mature embryos were transferred to germination medium with 1/3 MS macroelements and a 10 g/L sucrose supplement. After 12 weeks, 17 independent shoots (35.4% shoot germination) were obtained, corresponding to embryos that had formed either a single shoot or a shoot and a root (**Figure 1G**). Shoots from different germinated embryos were cultured individually on RP medium to enhance development of axillary shoots.

### Microsatellites Assay

**Figure 2:**
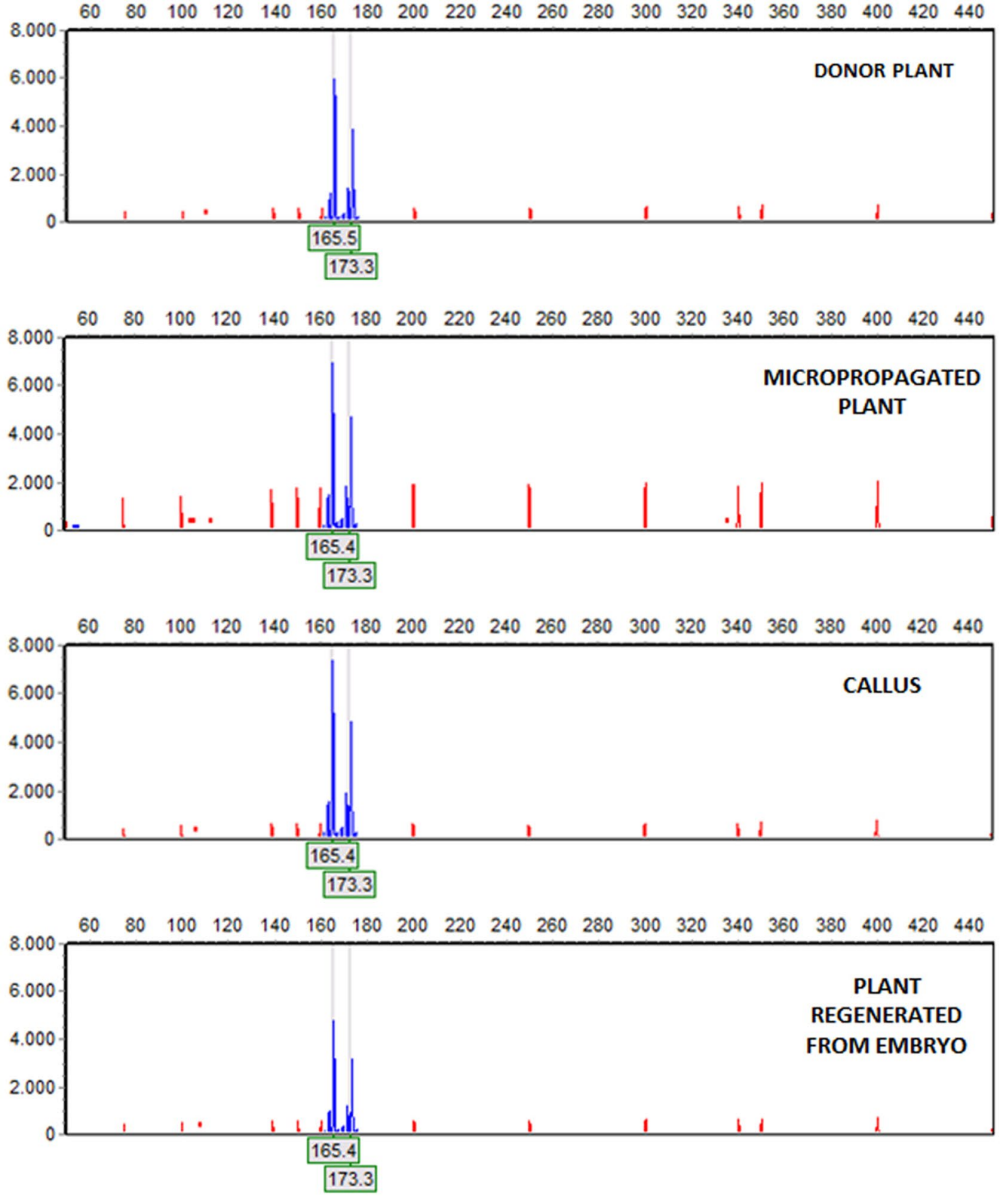
Amplification of the (6-FAM™)ssrOeUA-DCA09 in material of StopVert genotype. Electropherograms, from top to bottom: donor plant, a micropropagated plant, a piece of embryogenic callus derived from shoot apex and a plant regenerated from a somatic embryo. Electropherograms showed heterozygous individuals with two alleles, being the fragments size 165 and 173 bp.

After amplification, fragments generated from all materials were compared to the mother plant. The five SSR markers produced six alleles or fragments. Band size ranged between 138 and 236 pb (**Table 4**), being similar to the values described by De la Rosa et al. (2002) and Sefc et al. (2000) for olive. Samples of embryogenic callus, shoots regenerated from this callus and control shoots used as explant source, were monomorphic when compared to the mother plant for each primer pair. All analyzed markers were homozygous, except for AJ279859, which was heterozygous (**Table 4**). Electropherograms for SSR ssrOeUA-DCA09 for each type of sample analyzed are shown in **Figure 2**. The genetic stability analysis using these molecular markers showed that the genetic fidelity was 100% with respect to the mother plant.

**Table 4:** Allele size obtained with the amplification of five microsatellite markers in StopVert genotype.

Locus	Allele size (bp)			
	Donor plant	Axillary shoots *in vitro*	Embryogenic callus	Plants regenerated from somatic embryos
AJ279854	236	236	236	236
AJ279859	165/173	165/173	165/173	165/173
AJ279867	172	172	172	172
AJ416322	138	138	138	138
AJ416323	195	195	195	195

### RAPDs Assay

**Figure 3:**
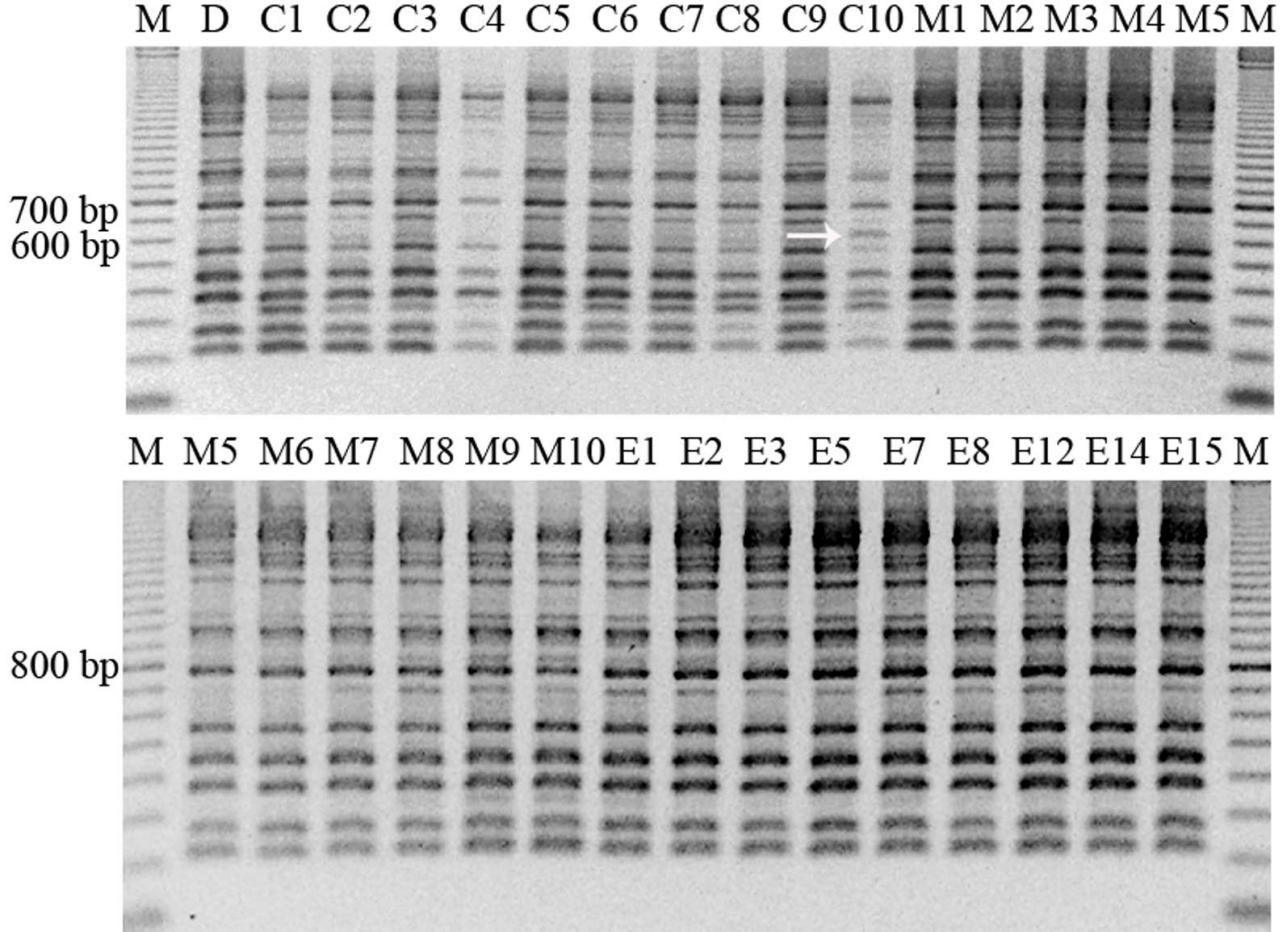
RAPD pattern obtained with the primer A1 in different samples of material of StopVert genotype, corresponding to D (donor plant), C1–C10 (callus samples from embryogenic callus from shoot apex), M1–M10 (micropropagated shoots derived from axillary buds of donor plant), and E1–E15 (plants regenerated from somatic embryos). The arrow shows the sample with a different band pattern. M = marker (100–2000 bp).

In this analysis, all samples previously studied were included, except regenerated plant from embryo number 4, since with this sample attempts to get PCR amplification were unsuccessful. RAPD profiles were generated using primers A1, B7, B15, E19, and F10, which amplified a high number of bands. A total of 50 bands were obtained, ranging the number of bands obtained per amplification between 9 and 11, and their size between 200 and 1,800 bp (**Table 5**). The genetic stability analysis with these five RAPD markers showed that shoots regenerated from somatic embryos and control shoots used as explant source presented the same band pattern than that of the mother plant (**Figure 3**). However, an additional band of 650 bp was amplified in a callus sample with primer A1 (**Figure 3**), indicating a level of polymorphism of 0.2% in the samples of the callus analyzed. Considering all samples, the variation rate was 0.07%.

**Table 5:** Number of scored bands and size band range (bp) with the amplification of five RAPDs markers in StopVert genotype.

Primers	N° of bands amplified	Size of band (bp)
A1	11	200-1600
B7	10	250-1600
B15	11	500-1800
E19	9	300-1300
F10	9	250-1400

## Discussion

### Somatic Embryogenesis Induction and Plant Regeneration From Adult Material in Wild Olive

Somatic embryogenesis has previously been observed in a few number of cultivated (Rugini and Caricato, 1995; Mazri et al., 2013; Toufik et al., 2014) and wild olive (Capelo et al., 2010) genotypes using adult explants. In general, embryogenic capacity is lower in mature explants than in those of juvenile origin. Cells of adult explants are less prone to dedifferentiation and reprogramming processes than their juvenile counterparts. Some authors pointed out that recalcitrance could have either a genetic (von Arnold, 2008) or epigenetic basis (Gahan, 2007). In this research, we have evaluated the embryogenic response of adult material from four wild olive genotypes, using two different induction protocols and different types of explants.

Genotype, type of explant, mineral formulation, and auxin/cytokinin ratio are key factors in embryogenic induction in adult material. In our case, embryogenic response was only observed in two out of the four selections evaluated. Along this line, Correia et al. (2011) pointed out the need to optimize culture conditions for a determined selection, since several genotypes could respond in a different manner. However, genotypes with a high embryogenic potential have less nutritional and hormonal requirements than those with low embryogenic capacity.

Type of explant plays a key role in embryogenic induction. Leaf sections (Corredoira et al., 2015) and shoot apex (San-José et al., 2010; Corredoira et al., 2015) of adult origin have been used in other woody species. In cultivated and wild olive, leaf and petiole explants had been recommended (Rugini and Caricato, 1995; Capelo et al., 2010; Mazri et al., 2013; Toufik et al., 2014); however, in this research, following comparison of three explant types, leaf, petiole, and shoot apex, embryogenic calli were only obtained when shoot apices were used, confirming previous observations of Corredoira et al. (2015) in eucalyptus. These authors indicated that shoot apex and leaves from the first node would be the most effective explants for somatic embryogenesis induction due to their high proliferation capacity and low differentiation stage.

Somatic embryogenesis induction generally requires addition of auxin and sometimes, cytokinin, to the culture medium. After hormonal treatment, some cells acquire embryogenic capacity being expressed in a medium without or with lower auxin concentration. In juvenile material from cultivated (Mitrakos et al., 1992) and wild olive (Orinos and Mitrakos, 1991), embryogenic induction process was achieved using a high auxin/cytokinin ratio (25 µM IBA–2.5 µM 2iP). In adult olive, positive results have been reported using either a low (Rugini and Caricato, 1995; Mazri et al., 2013) or a high (Capelo et al., 2010) auxin/cytokinin ratio. In our case, we only obtained embryogenic callus when using a high concentration of cytokinin (TDZ) and a low concentration of auxin (NAA), as reported by Mazri et al. (2013). In species, such as *Arachis hypogaea*, TDZ was more effective than auxin to induce direct somatic embryogenesis (Murthy et al., 1995). This hormone has been related to the modification of endogenous auxin synthesis (Murthy et al., 1998); probably, in adult olive, an increase in endogenous auxin levels could have occurred; i.e., auxin increases have been shown to play a key role in embryogenic induction in other systems (Rodríguez-Sanz et al., 2014; Fehér, 2015; Corredoira et al., 2017). Interestingly, in *Pelargonium* × *hortorum*, presence of auxin biosynthesis inhibitors in a TDZ supplemented medium interfered with induction of the embryogenic process (Hutchinson et al., 1996). In any case, additional studies are needed to elucidate the mechanisms of action of TDZ (Guo et al., 2011). Percentages of embryogenic response in adult material are variable and, in general, lower than those observed for juvenile explants. Mazri et al. (2013) reported about 10% response in cv. Dahbia, whereas in wild olive, the obtained percentages were in the range 2.28–27.6% (Capelo et al., 2010). In this research, embryogenic induction rates of 2.5% and 5% were obtained for Ac-18 and StopVert genotypes, respectively. Moreover, the latter showed a much better proliferation capacity of the embryogenic callus, confirming the important effect of genotype.

Using the protocol developed by Cerezo et al. (2011), somatic embryos from StopVert genotype could be maturated and converted to plants. The percentage of shoot germination, 35.4%, was lower than that reported by Cerezo et al. (2011) for somatic embryos derived from radicle explants (55%). In adult olive, only Rugini and Caricato (1995) were able to regenerate plants from cvs. Canino and Moraiolo. To our knowledge, this is the first report where plant regeneration *via* somatic embryogenesis has been obtained in adult material from wild olive.

### Regenerated Plants of the Wild Olive Genotype StopVert are Genetically Stable

The *in vitro* regeneration system plays a key role in the genetic stability of the material obtained. In olive plants, of juvenile origin, regenerated *via* somatic embryogenesis, contradictory results have been observed, i.e., Shibli et al. (2001) did not observe any changes in morphology, whereas Bradaï et al. (2016) reported a strong effect of genotype and time in culture in the appearance of variations such as fasciated shoots, three shoots per whorl, or double leaves. Leva (2009) obtained two variant phenotypes, BOS (shrubby) and COS (columnar), from somatic embryos derived of cotyledon explants. These results confirm previous observations of Karp (1994) and Wang and Wang (2012), about the important effect of genotype and time in culture in the occurrence of SV. In this investigation, morphological alterations were not observed in regenerated plants derived from somatic embryos of StopVert genotype.

Molecular markers have been widely used as a complementary tool to detect somaclonal variation in regenerated plants. Lopes et al. (2009) did not find variations between embryogenic material and the mother plant in *O. europaea*, as well as in *O. maderensis*, using SSR markers. Similarly, in this investigation, no SSR variations were detected when comparing embryogenic callus and shoots regenerated from this callus with the controls. Contrary to these results, a low rate of variation in the embryogenic callus was obtained when using RAPD markers. All RAPD profiles generated were monomorphic, except for one callus sample, which amplified an additional 650 bp band with primer A1, absent in the rest of samples. Variations detected by RAPD markers in callus but not in the regenerated plants have also been found in other species such as *Picea glauca* (De Verno et al., 1999). In cork oak (Lopes et al., 2006), a high level of polymorphism was obtained using SSR markers in somatic embryos but not in regenerated plants. Some authors have proposed that this could occur due to *de novo* mutations during the differentiation–dedifferentiation process *in vitro* (Jain, 2001). Thus, embryogenic callus would be formed by a mix of stable and unstable cells, but only cells with an unaltered genome could regenerate plants (De Verno et al., 1999; Miñano et al., 2014). In this research, since regenerated plants showed uniformity with both molecular markers, it can be suggested that the protocol used to induce somatic embryogenesis from adult material in wild olive did not produce genetic alterations that could be detected by SSR and RAPD markers. Despite this fact, total genetic fidelity is not possible to assure since these techniques analyze only a piece of genome. Moreover, further molecular analysis as well as a study of true-to-type phenotype of regenerated plants would be recommended, especially if embryogenic callus were kept for a long time in culture. In any case, taking into account that commercial olive micropropagation shows a strong genotype effect (Zuccherelli and Zuccherelli, 2002), regeneration and genetic fidelity of adult plants derived from somatic embryos open new opportunities to propagate recalcitrant cultivars, i.e., obtained material could be used as revitalized mother plants and further multiplied either *in vitro*, through nodal segmentation of elongated shoots, or *in vivo*, by rooted minicuttings, as reported in other woody species (Martínez et al., 2012; Georget et al., 2017).

### Conclusions

In this research, somatic embryogenesis has been induced from adult material of two wild olive genotypes, StopVert and Ac-18, highly resistant to D *V. dahliae* pathotype. The type of explant played a key role in the process, being the shoot apex the most responsive. Plants from somatic embryos of StopVert genotype have also been regenerated and molecular analysis using SSRs and RAPDs markers confirmed genetic fidelity of the recovered plants. As far as we know, this is the first time that plants have been regenerated *via* somatic embryogenesis using adult explants of wild olive. The protocol described in this research will open the door to propagate recalcitrant olive genotypes; in addition, it will allow the development of biotechnological tools, such as genetic transformation and gene editing, and their application to study the genetic mechanisms underlying resistance to D *V. dahliae* pathotype in these wild olive genotypes, with the final purpose of introducing this trait into cultivated olive.

## Data Availability Statement

The datasets generated for this study are available on request to the corresponding author.

## Author Contributions

IN was responsible for *in vitro* regeneration experiments. IN and CM carried out the molecular analysis. RJ-D developed the wild olive resistant genotypes used in this study and revised the manuscript. JM and FP-A planned this research, designed the experiments, and wrote the manuscript.

## Funding

This investigation was funded by Junta de Andalucía (Grant No. P11-AGR-7992) and by the Ministerio de Ciencia e Innovación of Spain and Feder European Union Funds (Grant No. AGL2017-83368-C2-1-R).

## Conflict of Interest

The authors declare that the research was conducted in the absence of any commercial or financial relationships that could be construed as a potential conflict of interest.
