# Supplementary material for: Plant Regeneration via Somatic Embryogenesis in Mature Wild Olive Genotypes Resistant to the Defoliating Pathotype of Verticillium dahliae
Source: Front Plant Sci. 2019 Nov 14;10:1471. doi: 10.3389/fpls.2019.01471 (PMC6868069; doi:10.3389/fpls.2019.01471)

Plant regeneration via somatic embryogenesis in mature wild olive genotypes resistant to the defoliating pathotype of *Verticillium dahliae*

Narváez, I., Martín, C., Jiménez-Díaz, R.M., Mercado, J.A., Pliego-Alfaro, F.

Supplementary material

Supplementary Fig. 1. Material of StopVert genotype used in this study (A-B) Donor plant in the greenhouse. (C) *In vitro* grown shoot originally derived from axillary buds of donor plant used as explant source. Bar in C corresponds to 1 cm.

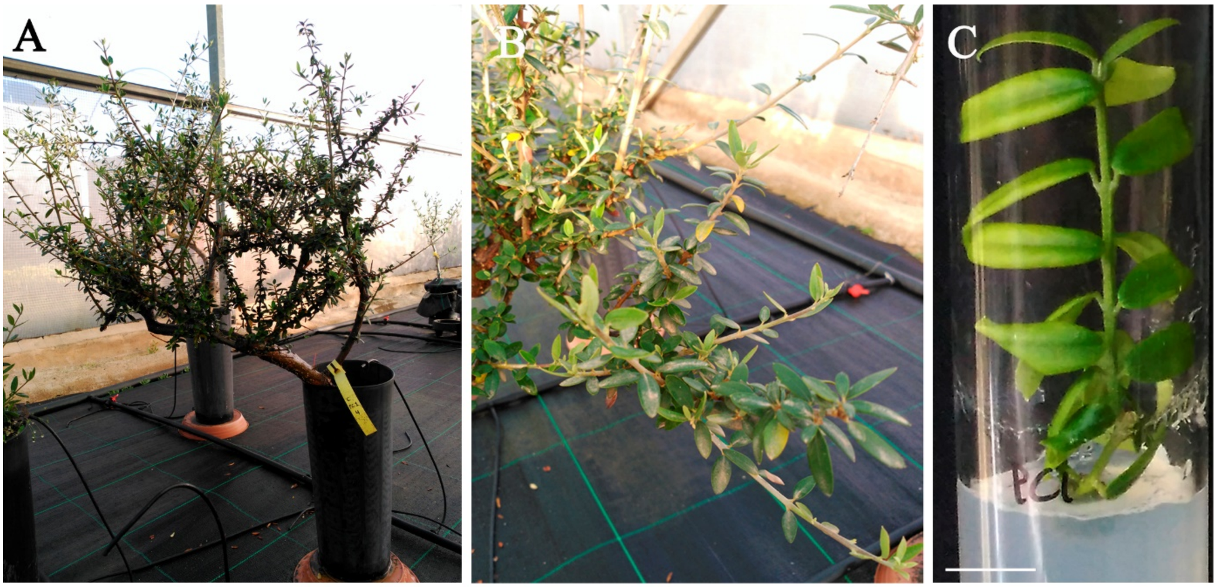

Supplementary Fig. 2. Arbitrary scale based on visual criteria used to estimate the amount of callus formed on basal part of leaves after 4 weeks on basal ½ MS medium. From left to right: initial explant; (1) < 40% explant surface covered by callus; (2) 40-80% explant surface covered by callus; (3) 80-100% explant surface covered by callus. Bars correspond to 0.5 cm.

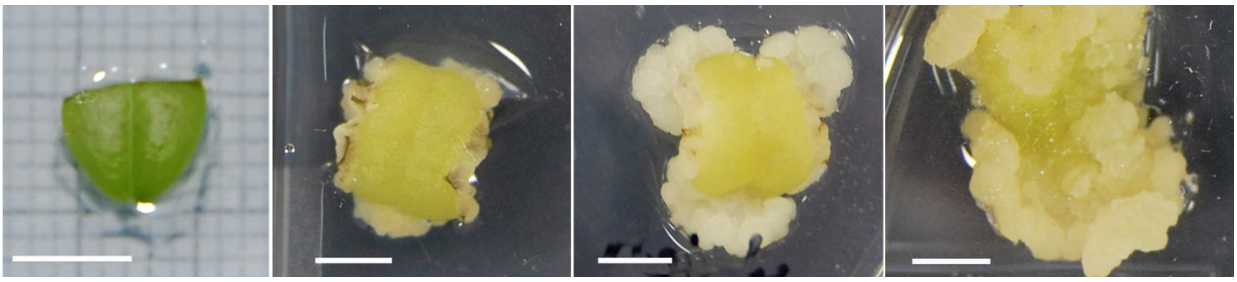

Supplement: Supplementary file 1 [file DataSheet_1.pdf]
